# Dynamic Optics with Transparency and Color Changes under Ambient Conditions

**DOI:** 10.3390/polym11010103

**Published:** 2019-01-09

**Authors:** Yejia Jiang, Songshan Zeng, Yu Yao, Shiyu Xu, Qiaonan Dong, Pingxu Chen, Zhaofeng Wang, Monica Zhang, Mengting Zhu, Gefan Xu, Huidan Zeng, Luyi Sun

**Affiliations:** 1 Key Laboratory for Ultrafine Materials of Ministry of Education, School of Materials Science and Engineering, East China University of Science and Technology, Shanghai 200237, China; y45160041@mail.ecust.edu.cn (Y.J.); 10161443@mail.ecust.edu.cn (Y.Y.); 10161447@mail.ecust.edu.cn (S.X.); 10161462@mail.ecust.edu.cn (Q.D.); 2 Polymer Program, Institute of Materials Science and Department of Chemical & Biomolecular Engineering, University of Connecticut, Storrs, CT 06269, USA; songshan.zeng@uconn.edu (S.Z.); monica.zhang@uconn.edu (M.Z.); mengting.zhu@uconn.edu (M.Z.); gefan.xu@uconn.edu (G.X.); 3 National Engineering Laboratory of Plastics Modification and Processing, and Research and Development Center, Kingfa Science and Technology Company, Ltd., Guangzhou 510663, China; pinger@kingfa.com; 4 State Key Laboratory of Solid Lubrication, Lanzhou Institute of Chemical Physics, Chinese Academy of Sciences, Lanzhou, Gansu 730000, China; zhfwang@licp.cas.cn

**Keywords:** mechanochromism, elastomer, silica, color, transparency

## Abstract

Mechanochromic materials have recently received tremendous attention because of their potential applications in humanoid robots, smart windows, strain sensors, anti-counterfeit tags, etc. However, improvements in device design are highly desired for practical implementation in a broader working environment with a high stability. In this article, a novel and robust mechanochromism was designed and fabricated via a facile method. Silica nanoparticles (NPs) that serve as a trigger of color switch were embedded in elastomer to form a bi-layer hybrid film. Upon stretching under ambient conditions, the hybrid film can change color as well as transparency. Furthermore, it demonstrates excellent reversibility and reproducibility and is promising for widespread application.

## 1. Introduction

Mechanochromic materials are of great significance because of their unique color change properties [1]. They show reversible and controlled color changes by mechanical stimuli [2] and have attracted tremendous interests attributed to their widespread application in humanoid robots [3], smart windows [4,5], anti-counterfeit tags [6], varistor [7,8,9], etc. However, their commercialization remains challenging because of the experimental complexity in material structure design and fabrication. In fact, the phenomenon of color change as stimulated by an external stimuli is common in nature. For example, octopuses are able to instantaneously change their skin color for the purpose of camouflage [10]. Chameleons have an amazing “pigment store” in their skin, which can change color according to the surrounding light, temperature, and living environment [11]. Inspired by these intriguing phenomena, researchers have fabricated a series of materials that are able to change their optical properties by molecular design and modification [12,13] or material construction [6,12,14] as triggered by light [15], electrical field [16], temperature [17], swelling [18], and mechanical strain [14,19,20].

Silica nanoparticles (NPs) are known to form an amorphous structure to generate non-iridescent structural colors [21,22,23]. In most of the previous efforts, black materials with an excellent light absorption ability were mixed to prevent the amorphous structure’s strong incoherent-light scattering across the entire visible spectrum. Ge et al. [5] developed a robust smart window by embedding silica NPs into an elastomer to exhibit angle-independent color upon stretching. However, achieving reliable and apparent color transformation in daily environment is much more challenging. Most of the devices show poor chemical stability and/or reversibility [24,25], or require UV radiation [14], or require mechanical grinding [12,13], which significantly limit their applications in daily life.

Herein, we design a new mechanochromism, which can instantaneously change color under ambient conditions with excellent reversibility. The hybrid film has a bilayer structure, in which an amorphous array of silica nanoparticles is embedded at the bottom of a polydimethylsiloxane (PDMS) elastomer containing a dye. The hybrid film exhibits amber under ambient conditions (under natural light at room temperature). Upon being stretched, the device exhibits a gradually lowered transmittance and displays a pinkish purple color at a strain of 80% or higher. Moreover, the mechanochromic effect of the hybrid film is highly reproducible even after over 1100 stretching-releasing cycles and is promising for widespread application.

## 2. Experimental

### 2.1. Materials

Tetraethyl orthosilicate (TEOS, 98%, Yonghua Chemical Technology, Shanghai, China), isopropanol (IPA, 99.8%, Macklin, Shanghai, China), ammonium hydroxide (~28–30%, GreaGent, Shanghai, China), ethanol (99%, GreaGent, Shanghai, China), PDMS (Sylgard-184, Dow Corning, Midland, MI, USA), and Rhodamine B (99%, Adamas, Basel, Switzerland) were used as received. 

### 2.2. Synthesis of Silica NPs

Monodispersed SiO_2_ NPs were prepared by using a modified Stöber method [21,26]. In the process to synthesize silica NPs, 50.0 mL ethanol-based solution containing 4.5 mL TEOS was added to 50.0 mL water solution containing 9.0 mL aqueous ammonia. After 2 h of stirring at 30 °C, the resulting silica NP suspension was centrifuged at a relative centrifugal force (RCF) of 10,397× *g* for 10 min, followed by ultrasonication in an isopropanol solution. The above process was repeated at least 3 times until the solution became neutral.

### 2.3. Fabrication of Hybrid Films

The silica NPs were dispersed in isopropanol at 10.0 wt % and ultrasonicated for 2 h, forming a uniform dispersion. An airbrush (IWATA HP-C plus, Yokohama, Japan) with a nozzle size of 0.3 mm was used to spray the above dispersion onto a polystyrene petri dish, which serves as the foundation. After spraying 8 times, a liquid PDMS (base to curing agent ratio = 10:1) with Rhodamine B (Rhodamine B concentration: 4.8 × 10^−5^ mol·g^−1^) was then cast atop the silica NP layer and then cured at 65 ºC for 4 h to prepare the hybrid film. 

### 2.4. Characterization

The morphology of the SiO_2_ NP layer was characterized using a field emission scanning electron microscope (FESEM, HITACHI S-4800, Tokyo, Japan) with an accelerating voltage of 10 kV. The particle size and size distribution of the SiO_2_ NPs were analyzed using dynamic light scattering (DLS, Malvern Zetasizer Nano ZS90 analyzer, Malvern, UK). The diffraction patterns were captured using a He–Ne gas laser (633 nm, 1 mW, Nanjing Pudong Laser Technology Research Institute, Nanjing, China) in a dark room. 

The hybrid film was mounted on a custom-built stretching tool to characterize its color change property with an altering strain. The reflectance of the hybrid film was recorded on a UV-Vis-NIR spectrophotometer (Shimadzu UV-3600, Kyoto, Japan). All of the digital photos were taken by an iPhone 8 Plus. Cyclic fatigue tests of the samples were conducted on a CTM 2500 universal testing machine. 

## 3. Results and Discussion

**Figure 1:**
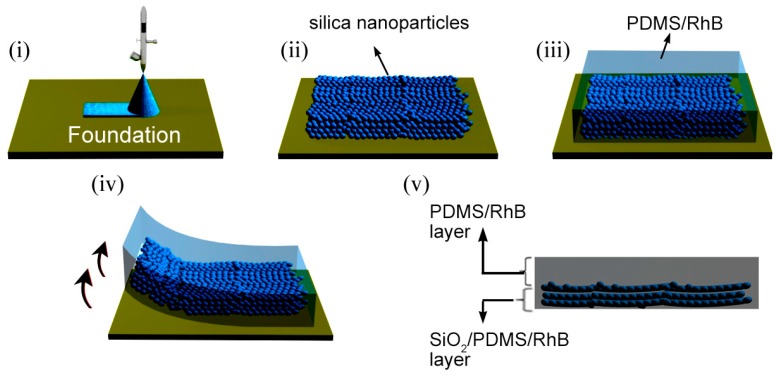
Schematic of the fabrication process of the SiO_2_/PDMS/RhB hybrid film. (**i**) Spray coating silica NPs onto a foundation; (**ii**) formation of a layer of amorphous silica NP arrays; (**iii**) casting PDMS precursor and Rhodamine B atop the silica NP layer; (**iv**) peeling off the hybrid film from the foundation after curing; (**v**) structure of the prepared SiO_2_/PDMS/RhB hybrid film.

The procedures to fabricate the hybrid film are illustrated in Figure 1. First, the silica NP dispersion was sprayed onto the foundation (i), leading to the formation of a layer of amorphous NP arrays with a thickness of ca. 3.4 μm upon drying (ii). Then, the homogenized liquid of PDMS precursor and Rhodamine B (RhB) was cast atop the silica NP layer and allowed it to settle (this step usually takes more than 12 h) (iii). After curing for 4 h at 65 °C, the hybrid film was peeled off from the foundation (iv). The final silica NPs/PDMS/Rhodamine B (SiO_2_/PDMS/RhB) bi-layer hybrid film was controlled to have a thickness of ca. 1 mm (v). 

**Figure 2:**
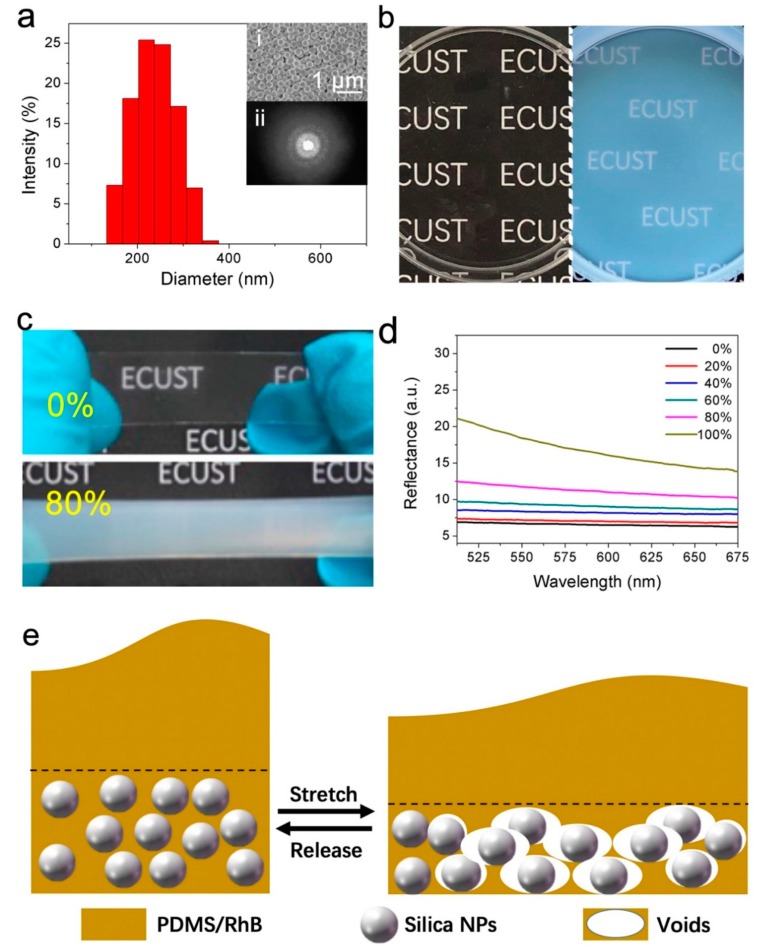
(**a**) Size distribution of the prepared silica NPs from dynamic light scattering. The inset (i) shows the SEM image of the spray-coated amorphous silica NP layer and (ii) shows a diffraction pattern of the coating layer using a 633 nm He-Ne laser; (**b**) A digital photo of a clean Petri dish (left) and a Petri dish coated with the amorphous silica NP layer, exhibiting a pale white color (right); (**c**) Digital photos of un-stretched (top) and stretched (bottom, 80% strain) SiO_2_/PDMS film; (**d**) Reflectance UV spectra of the SiO_2_/PDMS at various strains; (**e**) Schematic to demonstrate void formation upon stretching and returning to the original state when released.

Silica NP colloidal arrays can form an amorphous structure to generate non-iridescent structural colors, which could cause a pale appearance. Herein, we designed to intentionally take advantage of such a phenomenon by fabricating amorphous silica NP arrays, which exhibit a pale white color under naked eyes. Virtually monodispersed silica NPs were synthesized (Figure 2a), whose average diameter was ca. 233 nm, consistent with the result from the SEM characterization. Their polydispersity index (PDI) was only 0.8%, indicating that the NPs are close to be monodisperse [27].

After spray coating the silica NP dispersion on a polystyrene petri dish foundation, an amorphous structure formed as shown in Figure 2a(i). The diffraction pattern (Figure 2a(ii)) captured by a He–Ne laser further proved that the structure of the silica NP layer was short-range ordered and isotropic. When multiple cycles of coating were applied onto the foundation, a whitish surface formed (Figure 2b), which is in accordance with its microstructure.

When casting the PDMS precursor solution over the NP arrays, the voids in-between the NPs were filled [5]. The resultant SiO_2_/PDMS film is highly transparent at an un-stretched state (Figure 2c (top)) because of the similar refractive indexes of PDMS (1.425 at 632.8 nm) [28] and silica (1.457 at 632.8 nm) [29], but becomes opaque and appears a pale white color (Figure 2c (bottom)) at ca. 80% strain. The corresponding spectroscopy characterization results further proved the phenomena we observed. As shown in Figure 2d, the spectra remained virtually flat from 0 to 60% strain, while an increase of scattering appeared from 80% strain in visible and near infrared (Vis-NIR) range. This increasing of scattering indicates that a pale white color can be generated in SiO_2_/PDMS upon stretching [23].

Silica NPs play an important role in triggering color/transparency change as shown in Figure 2c. After liquid PDMS was cast atop the silica NP layer, the gaps among NPs were impregnated by the liquid after hours of settling. When the cured SiO_2_/PDMS film is stretched, nano-voids formed between PDMS and silica NPs [5]. The arrangement of the voids formed locally should also be amorphous, analogous to the fabricated amorphous silica NP arrays (Figure 2a(i)). As a result, we can observe the pale white color similar to the silica NP layer.

**Figure 3:**
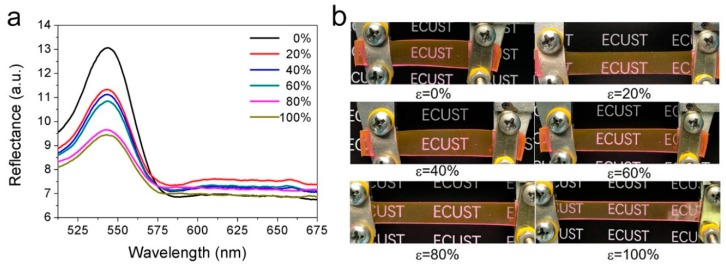
(**a**) Reflectance spectra and (**b**) digital photos of the PDMS/RhB at various strains.

Rhodamine B is extensively used as a laser dye [30] and it can be uniformly dispersed into PDMS to form a luminescent layer [14]. A soft PDMS film containing Rhodamine B (PDMS/RhB) was also prepared. The reflectance spectra of the PDMS/RhB film are displayed in Figure 3a. There was only one peak at ca. 544 nm, characteristic of Rhodamine B [31]. The overall appearance of the PDMS/RhB film at various strains is shown in Figure 3b. With an increasing strain, the peak remained at the original wavelength but its intensity decreased gradually. This is expected as the film became thinner while there were no additional factors to trigger color change [32].

**Figure 4:**
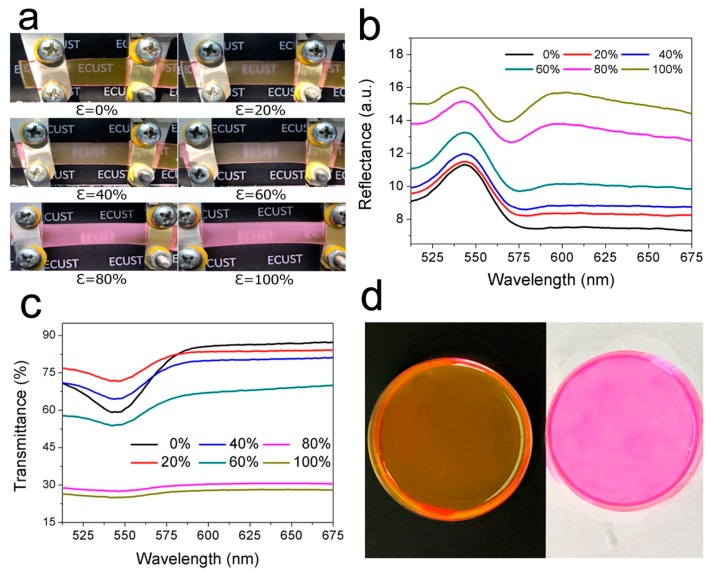
(**a**) Digital photographs; (**b**) reflectance spectra; and (**c**) transmittance spectra of the SiO_2_/PDMS/RhB film at various strains; (**d**) A digital photo of RhB ethanol solution on a black (left) and white background (right).

By simply combining the above two systems, we managed to create a novel mechanochromic device by casting a liquid PDMS containing RhB to the silica NP layer. Figure 4a shows the digital photographs of the resultant SiO_2_/PDMS/RhB hybrid film at various strains. Viewing by naked eyes under ambient conditions, the film exhibits the characteristic amber color of Rhodamine B at an un-stretched state. The color alteration from amber to pinkish purple occurred at ca. 80% strain and demonstrated a deeper pink at about 100% strain. Moreover, the hybrid film exhibits a change of transparency upon being stretched. It changes from transparent to translucent at ca. 20% strain and completely opaque at ca. 100% strain.

The corresponding reflectance spectra of the SiO_2_/PDMS/RhB hybrid film at various strains are shown in Figure 4b. In a released state, only one peak appeared in the visible spectrum range at ca. 544 nm, which was very similar to the reflectance spectrum of the un-stretched PDMS/RhB as shown in Figure 3a. With an increasing strain, the spectrum of the hybrid film only changed marginally until the strain reached 80%. This is because of the formation of nano-voids on both sides of the silica NPs under this strain due to the poor interfacial adhesion between the stretchable hydrophobic PDMS and the hydrophilic silica NPs. This result is not only consistent with the observation discussed above, but also is in accordance with the largest drop in the transmittance of the hybrid film upon stretching up to 80% (Figure 4c), which indicates that the void formation reduces opacity and further affects the color change phenomenon. From 80% strain, a broad peak in the Vis–NIR range appeared besides the characteristic peak at ca. 544 nm, similar to the trend of the SiO_2_/PDMS control sample (Figure 2d). With an increasing strain, the intensity of the peak at ca. 544 nm gradually decreased while the broad peak became more intensive, reaching a comparable intensity as the one at 544 nm at ca. 100% strain. This suggests that the overall color should be determined by the combination of the amber color caused by Rhodamine B and the pale white color caused by the gaps in between the PDMS matrix and the silica NPs. Further, both Rhodamine B and silica NPs are responsible for the mechanochromic property of the hybrid film.

Based on the observations and characterizations, the color change mechanism of the SiO_2_/PDMS/RhB hybrid film is proposed as follows. According to the reflectance spectra of the PDMS/RhB at various strains (Figure 3a), the only peak at ca. 544 nm represents the wavelength of a yellow-green color [33]. The slight red light reflected from RhB and yellow-green color mixed to produce the amber color we observed. When being stretched, the pale white color caused by SiO_2_/PDMS is similar to a white background. With the increase of tensile elongation, the increasing intensity of the white color caused the red light selectively reflected by PDMS/RhB to dramatically increase, forming the pinkish purple color we observed later. To simulate this phenomenon, we charged a RhB ethanol solution in two clean Petri dishes, one on a black background and the other on a white background, as shown in Figure 4d. As one can observe, the color displayed is basically in line with our experimental results, which further supports the explanation we proposed. 

**Figure 5:**
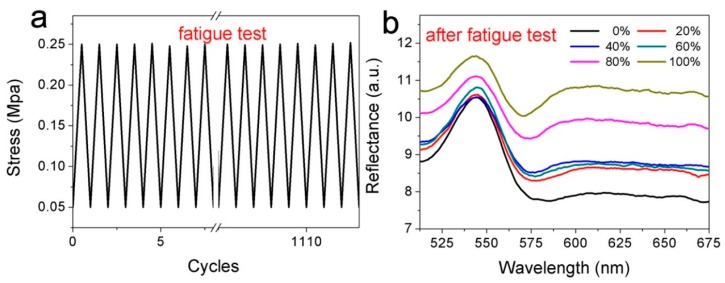
(**a**) Stress as a function of cycles of strain; (**b**) reflectance spectra of the SiO_2_/PDMS/RhB hybrid film at different strains after the fatigue test.

To further investigate the mechanochromic behaviors of the hybrid film subjected to cyclic loading, we repeatedly stretched the SiO_2_/PDMS/RhB hybrid film to 180% strain and released the force to allow the film to recover completely (Figure 5a). The stress remained virtually constant during the over 1100 cycles of fatigue test, which suggests that the structure of the silica NP layer was well maintained. After over 1100 cycles, we measured the reflectance spectra of the SiO_2_/PDMS/RhB hybrid film at various strains, and the results are shown in Figure 5b. The characteristic peaks also exhibited a similar change trend as the sample before the fatigue test (Figure 4b), which suggests that the hybrid film is robust and cyclic stable.

## 4. Conclusions

In summary, we fabricated a mechanochromic hybrid film that is composed of an array of silica NPs embedded at the bottom of a PDMS/RhB layer. The film can change its color from amber to pinkish purple through mechanical stretching and recovers back to its original color as released, during which it also changes from transparent to opaque and back to transparent. Moreover, the color and transparency change is highly reversible and reproducible. Most importantly, the color change phenomenon is visible under ambient conditions, which helps significantly broaden the applications of this mechanochromic film.
